# The Comprehensive Steroidome in Complete TSPO/PBR Knockout Mice under Basal Conditions

**DOI:** 10.3390/ijms24032474

**Published:** 2023-01-27

**Authors:** Philippe Liere, Guo-Jun Liu, Antoine Pianos, Ryan J. Middleton, Richard B. Banati, Yvette Akwa

**Affiliations:** 1 Disease and Hormones of the Nervous System, U1195 Inserm-Université Paris Saclay, 80 rue du Général Leclerc, 94276 Kremlin-Bicêtre, France; 2 Australian Nuclear Science and Technology Organisation (ANSTO), Kirrawee, NSW 2232, Australia; 3 Faculty of Medicine and Health, Medical Imaging Sciences, Brain and Mind Centre, University of Sydney, Camperdown, NSW 2006, Australia

**Keywords:** peripheral benzodiazepine receptor, PBR, translocator protein, TSPO, steroidogenesis, neurosteroids, endocrine glands, mitochondria, gas chromatography–tandem mass spectrometry, gene knockout

## Abstract

The 18 kDa translocator protein (TSPO/PBR) is a multifunctional evolutionary highly conserved outer mitochondrial membrane protein. Decades of research has reported an obligatory role of TSPO/PBR in both mitochondrial cholesterol transport and, thus, steroid production. However, the strict dependency of steroidogenesis on TSPO/PBR has remained controversial. The aim of this study was to provide insight into the steroid profile in complete C57BL/6-^Tspotm1GuWu(GuwiyangWurra)^-knockout male mice (TSPO-KO) under basal conditions. The steroidome in the brain, adrenal glands, testes and plasma was measured by gas chromatography coupled to tandem mass spectrometry (GC-MS/MS). We found that steroids present in wild-type (WT) mice were also detected in TSPO-KO mice, including pregnenolone (PREG), progestogens, mineralo-glucocorticosteroids and androgens. The concentrations of PREG and most metabolites were similar between genotypes, except a significant decrease in the levels of the 5α-reduced metabolites of progesterone (PROG) in adrenal glands and plasma and of the 5α-reduced metabolites of corticosterone (B) in plasma in TSPO-KO compared to WT animals, suggesting other regulatory functions for the TSPO/PBR. The expression levels of the voltage-dependent anion-selective channel (VDAC-1), CYP11A1 and 5α-reductase were not significantly different between both groups. Thus, the complete deletion of the tspo gene in male mice does not impair de novo steroidogenesis in vivo.

## 1. Introduction

**Figure 1:**
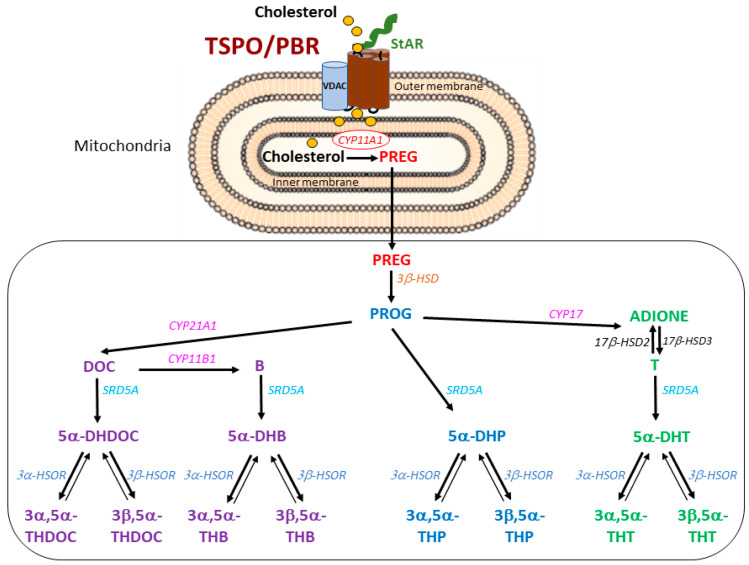
Cholesterol transport into the mitochondria and the enzymatic pathways of steroid synthesis from cholesterol in male mice. The current model involves the transport of cholesterol via StAR to the outer mitochondrial membrane of TSPO/PBR. Once cholesterol has entered mitochondria, it is converted by CYP11A1 into PREG, the precursor of all steroids. Progestogens (blue), mineralo-glucocorticoids (purple) and androgens (green) are the main families of steroids. PREG is further metabolized to PROG, the precursor of DOC and B. T is the major androgen formed from ADIONE, a direct product of PROG. PROG, DOC, B and T can then give rise to their respective 5α- and 3α/3β-reduced metabolites. Irreversible enzymatic reactions are denoted by a single plain arrow and reversible ones are shown by a double-headed plain arrow. The abbreviated names of steroidogenic enzyme are in italics. CYP: cytochrome P450 family (CYP11A1: P450 side chain cleavage, CYP17: 17β-hydroxylase,17–20 lyase, CYP21A1: 21α-hydroxylase, CYP11B1: 11β-hydroxylase), 3β-HSD: 3β-hydroxysteroid dehydrogenase-Δ5→Δ4 isomerase, SRD5A: 5α-reductase, HSOR: hydroxysteroid oxidoreductase, 17β-HSD: 17β-hydroxysteroid dehydrogenase. PREG: pregnenolone, PROG: progesterone, DOC: 11-deoxycorticosterone, B: corticosterone, DHP: dihydroprogesterone, THP: tetrahydroprogesterone, DHDOC: dihydrodeoxycorticosterone, THDOC: tetrahydrodeoxycorticosterone, DHB: dihydrocorticosterone, THB: tetrahydrocorticosterone, T: testosterone, DHT: dihydrotestosterone, THT: tetrahydrotestosterone, ADIONE: androstenedione. VDAC: voltage-dependent anion channel.

Steroid hormones and neurosteroids are produced in the endocrine glands and central nervous system, respectively, and are key endogenous pleiotropic molecules regulating fundamental functions. The synthesis of steroid hormones (progestagens, corticosteroids, androgens and estrogens) and their precursors and metabolites involves many biological processes. Briefly, steroids are produced either via de novo synthesis from cholesterol or through the local biosynthesis of steroid intermediates [1]. Amongst the first steps of steroidogenesis are the supply of cholesterol by the steroidogenic acute regulatory protein (StAR) [2,3] and the transport of cholesterol into the mitochondria (Figure 1). A large body of literature [4,5,6] has assigned the function of cholesterol transport across the mitochondrial inter-membrane space to a specific five-membrane-domain protein (Figure 1), previously known as the peripheral-type benzodiazepine receptor (PBR) and later renamed the 18 kDa translocator protein (TSPO) to signify its purported main role in the translocation of cholesterol as a vital obligatory initial step in the synthesis of steroids [4,7]. TSPO/PBR is a highly conserved protein located in the outer mitochondrial membrane [8] in a multimeric complex suggested to include the 34 kDa voltage-dependent anion channel (VDAC-1), the ATPase family AAA-domain containing protein 3A (ATAD3A) and the TSPO-associated protein 7 (PAP7, acyl-CoA-binding-domain 3 (ACBD3)) [9,10], though future research may reveal the involvement of other proteins. TSPO/PBR is ubiquitously expressed, predominantly in tissues synthesizing steroid hormones; i.e., adrenal glands and gonads [11].

The first and rate-limiting step of de novo steroidogenesis is the conversion of cholesterol into pregnenolone (PREG) by mitochondrial enzyme cytochrome P450 side chain cleavage (P450scc or CYP11A1) (Figure 1) [7]. The main steroidogenic pathways in male rodents are illustrated in Figure 1. 3β-hydroxysteroid dehydrogenase (3β-HSD) converts PREG to progesterone (PROG). PROG can then be converted to 11-deoxycorticosterone (DOC) by 21-hydroxylase (CYP21A1), which is metabolized to corticosterone (B) by 11β-hydroxylase (CYP11B1). PROG can also be converted to androstenedione (ADIONE) by 17α-hydroxylase/17,20 lyase (CYP17) followed by testosterone (T) by 17β-hydroxysteroid dehydrogenase (17β-HSD3). The 5α-reductase enzymes (SRD5A type 1 and type 2) convert PROG, DOC, B and T to their respective 5α-reduced metabolites, which are further converted to their 3α/β-reduced derivatives by 3α/β hydroxysteroid oxido-reductases (HSORs).

At the moment, the posited role for TSPO/PBR in steroidogenesis rests on the following assumptions and published data:

(i) TSPO is indispensable for the transfer of cholesterol into mitochondria and, thus, is a prerequisite for the generation of pregnenolone (PREG);
(ii) The identification of a consensus amino acid sequence for cholesterol recognition (CRAC) at the C-terminal region of TSPO/PBR [4,5];
(iii) Reduced plasma PREG concentration following tspo mutations in rats [6], reduced intratesticular and circulating testosterone levels in Tspo Amhr2-Cre-mediated global TSPO-KO mice [12] and unchanged levels of adrenal corticosterone following adrenocorticotropic hormone treatment in conditional TSPO-KO mice [13];
(iv) The ability of various TSPO/PBR agonists to enhance steroid production *in vitro* and in vivo in wild-type models [14,15,16,17].

Over the past decade, however, the function of TSPO in steroid synthesis has remained contentious, with genetic Tspo-deficient models failing to substantiate the role of TSPO/PBR in steroidogenesis and in embryonal viability [18]. Particularly, the extensive characterization of the complete C57BL/6-^Tspotm1GuWu(GuwiyangWurra)^-knockout (TSPO-KO) mouse model failed to reveal an impairment in the crucial rate of enzymatic conversion of cholesterol to PREG by CYP11A1 as a read-out of mitochondrial cholesterol transport or any other substantial pathological phenotype beyond a yet to be further characterized hematological variation [19]. Likewise, no significant sex-dependent variations; developmental or maturational changes or differences in the levels of protoporphyrin IX or the mRNA expression of steroidogenic acute regulatory (StAR) protein, CYP11A1 and Tspo2 in nine organs; or overt differences in the brain tissue response to neural injury were found [20,21,22,23]. In a commentary, Selvaraj et al. [24] have challenged the results obtained by Fan et al. [12] on reduced intratesticular and circulating testosterone levels in Amhr2-cre-mediated global TSPO-KO mice. The findings by [23] have also shown that the TSPO/PBR agonist PK-11195′s effect on steroidogenesis is not mediated by TSPO/PBR.

Presently, all published TSPO-KO models remain incompletely characterized with regard to de novo synthesis of PREG requiring the mitochondrial import of cholesterol and the extent to which steroid production in steroidogenic tissues may or may not show more subtle alterations due to the absence of TSPO/PBR. Most reports have been confined to the partial study of two to six steroids at a time only in the serum or plasma [6,13,19,21,22,25] and mostly using radioimmunoassays or enzyme-linked immunosorbent assays, except for two studies that used liquid chromatography coupled to mass spectrometry [6,25].

The main objective of the present work was, therefore, to undertake more comprehensive steroid profiling in the brain, adrenal glands, testes and plasma of adult male complete TSPO-KO mice [19] under basal conditions. The steroidome was established using the most reliable, sensitive and specific GC-MS/MS technology to identify and quantify simultaneously several steroids in tissues and fluids [26]. This analytical protocol made it possible to characterize progestogens, mineralo-glucocorticoids, androgens and estrogens, as well as their precursors and metabolites.

Based on the current model, if the absence of TSPO/PBR impacts steroidogenesis, it is expected that the de novo synthesis of PREG from cholesterol will be altered in each tissue and, therefore, the levels of subsequent metabolites will vary accordingly. Conversely, if the absence of TSPO/PBR does not affect PREG concentrations, any change in levels of steroids downstream from PREG production would point to other cell regulatory influences on the net activity of steroidogenic enzymes. In addition, we investigated whether Tspo gene deletion modulates compensatory translational mechanisms of proteins that may be functionally associated with TSPO/PBR for steroidogenesis. Therefore, we analyzed the levels of VDAC-1, the major component of the outer mitochondrial membrane [27]. We also investigated the protein expression levels of CYP11A1 and 5α-reductase (SRD5A1 or SDRA2) in the relevant tissues.

Our data show that PREG levels did not significantly change in the brain, adrenal glands, testes and plasma of TSPO-KO mice as compared to the WT, indicating that TSPO/PBR is not involved in the de novo synthesis of PREG. The concentrations of the other 22 detected steroids were similar in both genotypes, except for 5α-reduced metabolites of PROG in adrenal glands and plasma and 5α-reduced metabolites of B in plasma, which were reduced in TSPO-KO mice, providing some evidence that the TSPO/PBR has a limited indirect effect on steroidogenesis.

## 2. Results

For better clarity, steroids are presented according to their chemical class: (i) PREG, (ii) PROG and its 5α- and 3α/β-5α-reduced metabolites, (iii) 20α-reduced derivatives of PREG and PROG, (iv) B and DOC and their 5α- and 3α/β,5α-reduced metabolites and (v) ADIONE, T and its 5α- and 3α/β,5α-reduced metabolites.

### 2.1. Steroid Measurements in Wild-Type and TSPO-KO Mice under Basal Conditions

#### 2.1.1. Basal Concentrations of PREG

**Figure 2:**
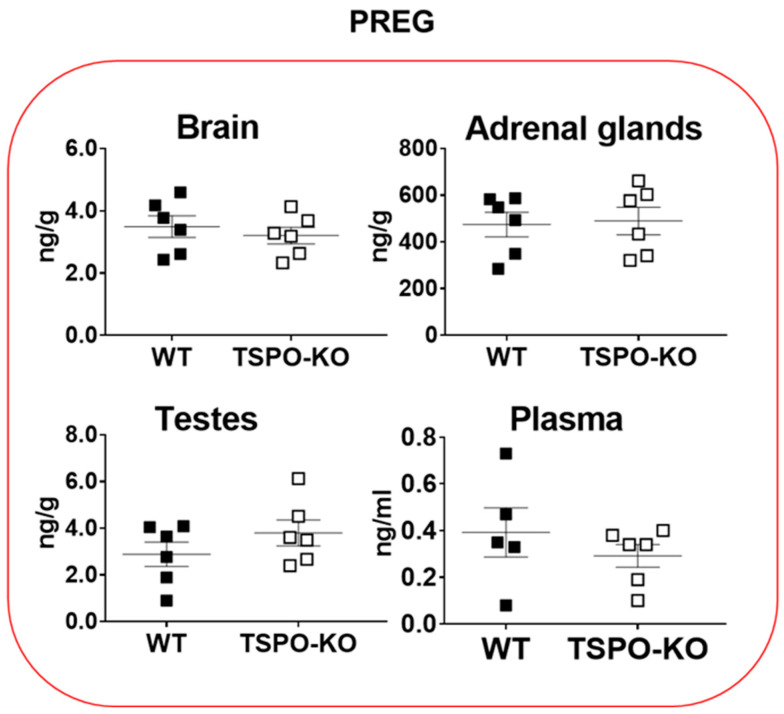
Concentrations of PREG in tissues of male WT and TSPO-KO mice analyzed by GC-MS/MS as described in the Material and Methods section. Data are expressed as means ± SEM and were analyzed by unpaired *t*-test (*n* = 5–6).

We investigated the ability of TSPO-KO mice to synthesize PREG de novo from cholesterol. As shown in Figure 2, PREG was present in all tissues. Its levels did not significantly change in the brain (*p* = 0.5264), adrenal glands (*p* = 0.8496), testes (*p* = 0.2606) and plasma (*p* = 0.3836) as compared to WT mice. The highest concentration of PREG was found in the adrenal glands in both groups and was, on average, 140 times higher than in the brain and testes and 1600 times higher than in plasma.

#### 2.1.2. Basal Concentrations of Progestogens

**Figure 3:**
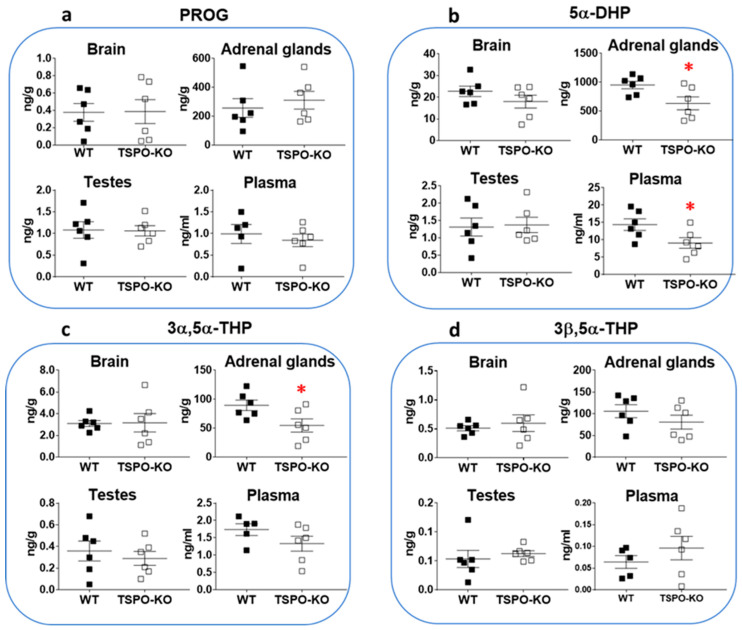
Concentrations of PROG, 5α-DHP, 3α,5α-THP and 3β,5α-THP in tissues of male WT and TSPO-KO mice analyzed by GC-MS/MS as described in the Material and Methods section. Data are expressed as means ± SEM and were analyzed by unpaired *t*-test (*n* = 5–6). * *p* < 0.05.

Levels of PROG and its 5α-reduced metabolites, 5α-dihydroprogesterone (5α-DHP), 3α,5α-tetrahydroprogesterone (3α,5α-THP or allopregnanolone) and 3β,5α-tetrahydroprogesterone (3β,5α-THP), were also measured in the same tissues. Figure 3 shows that these steroids were still produced in TSPO-KO mice.

PROG concentrations were not significantly altered after complete knockout of the tspo gene, irrespective of the tissue (Figure 3a; brain: *p* = 0.9600, adrenal glands: *p* = 0.5578, testes: *p* = 0.9361 and plasma: *p* = 0.5803). PROG levels in adrenal glands were on average 300–700 times higher than in other tissues in both WT and TSPO-KO mice.

Noticeably, in contrast to PROG, the 5α-DHP level was found to be significantly lower in adrenal glands of TSPO-KO mice than that in WT mice (33.4% decrease, *p* = 0.0347; Figure 3b). A significant decrease was also found for 5α-DHP levels in plasma (*p* = 0.0418) but not in the brain (although there was a 22% decrease *p* = 0.2449) or testes (*p* = 0.8641) of TSPO-KO mice as compared to WT.

The concentrations of 3α,5α-THP levels were also found to be significantly lower in adrenal glands of TSPO-KO mice than those in WT animals (39% decrease, *p* = 0.0363; Figure 3c). Differences in 3α,5α-THP levels between the two groups were not statistically significant in the brain (*p* = 0.9432), testes (*p* = 0.5557) and plasma (*p* = 0.1852). The highest concentrations of this steroid were measured in the adrenal glands of WT and TSPO-KO mice and were ~15 and 30 times higher than those in TSPO-KO and WT brains, respectively (Figure 3c). 3β,5α-THP was also detected at similar concentrations in WT and TSPO-KO mice (Figure 3d; brain: *p* = 0.5777, adrenal glands: *p* = 0.2802, testes: *p* = 0.5640 and plasma: *p* = 0.3503).

The enzyme 20α-hydroxysteroid dehydrogenase (20α-HSD) is known to metabolize PREG to 20α-dihydropregnenolone (20α-DHPREG) and PROG to 20α-dihydroprogesterone (20α-DHP) and to be localized in mice in several tissues, including the adrenal glands, testes and brain [28,29]. We measured low levels of 20α-DHPREG and 20α-DHP in all four tissues in both WT and TSPO-KO mice (Table 1). The levels of the 5α-reduced metabolite of 20α-DHP (5α,20α-THP) and those of the 3α- and 3β-reduced derivative of 5α,20α-THP (3α,5α,20α-HHP and 3β,5α,20α-HHP) were found at relatively high concentrations depending on the tissue and comparable in both genotypes. The highest concentrations of 5α,20α-THP were observed in the brain and adrenal glands, while 3α,5α,20α-HHP and 3β,5α,20α-HHP levels were higher in adrenal glands in each mouse group. No significant differences were noted between WT and TSPO-KO mice for any of these steroids regardless of the tissue.

#### 2.1.3. Basal Concentrations of Corticosteroids

**Figure 4:**
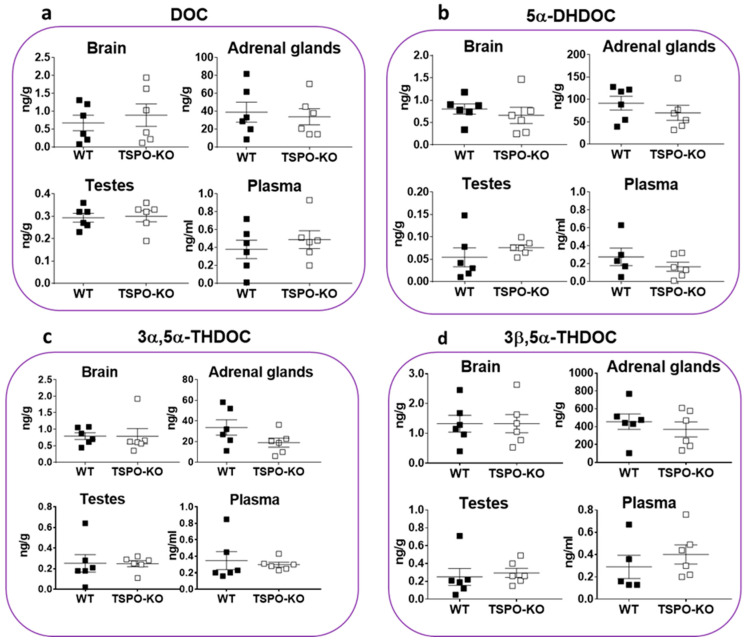
Concentrations of DOC and its 5α-reduced metabolites in tissues of male WT and TSPO-KO mice analyzed by GC-MS/MS as described in the Material and Methods section. Data are expressed as means ± SEM and were analyzed by unpaired *t*-test (*n* = 5–6).

Glucocorticosteroids are the class of steroid typically synthesized from the zona fasciculata of the adrenal cortex of vertebrates. 11-Deoxycorticosterone (DOC) is produced from PROG by 21α-hydroxylase (CYP21A1). As expected, its concentration was higher in adrenal glands as compared to other tissues and it did not significantly vary in TSPO-KO mice (Figure 4a, *p* = 0.7270). DOC levels were similar in the brain (*p* = 0.5809), testes (*p* = 0.8381) and plasma (*p* = 0.4679) in both groups.

We also quantified the 5α-reduced metabolites of DOC, 5α-dihydrodeoxycorticosterone (5α-DHDOC), 3α,5α-tetrahydrodeoxycorticosterone (3α,5α-THDOC) and 3β,5α-tetrahydrodeoxycorticosterone (3β,5α-THDOC) (Figure 4b–d, respectively). All were present at high concentrations in the adrenal glands and tended to decrease in the same organ in knockout mice, although not significantly (5α-DHDOC: *p* = 0.3676, 3α,5α-THDOC: *p* = 0.1193 and 3β,5α-THDOC: *p* = 0.4926). In the adrenal glands of both genotypes, the levels of 3β,5α-THDOC were ~tenfold higher than its 3α-stereoisomer and ~fourfold higher than that of its parent 5α-DHDOC (Figure 4d). In the brain and testes, the levels of 5α-DHDOC, 3α,5α-THDOC and 3β-5α-THDOC were relatively low (≤1 ng/g) and similar in WT and TSPO-KO mice. Their plasmatic concentrations were in the range of 0.2–0.5 ng/mL in both groups.

**Figure 5:**
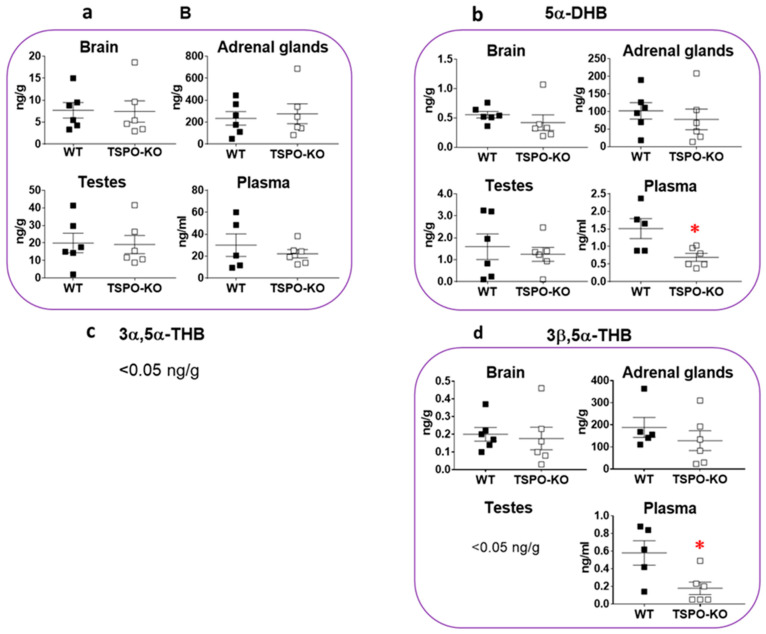
Concentrations of B and its 5α-reduced metabolites in tissues of male WT and TSPO-KO mice analyzed by GC-MS/MS as described in the Material and Methods section. Data are expressed as means ± SEM and were analyzed by unpaired *t*-test. * *p* < 0.05.

Corticosterone (B) is the major corticosteroid hormone synthesized in the adrenal glands from DOC by cytochrome 11β-hydroxylase (CYP11B). Accordingly, the highest concentrations of this steroid were measured in adrenal glands as compared to other tissues, but they were not significantly different between WT and TSPO-KO (*p* = 0.7059, Figure 5a). Adrenal levels of B were ~20 higher than in the brain and ~10 times higher than in testes and plasma in both genotypes. In other tissues, similar levels of B were measured in both genotypes (Figure 5a, brain: *p* = 0.9264, testes: *p* = 0.9106 and plasma: *p* = 0.4621).

The quantification of the 5α-reduced metabolites of B indicated that 5α-dihydrocorticosterone (5α-DHB) was predominantly produced in adrenal glands, as expected, at concentrations that were not significantly different between both genotypes (*p* = 0.5336, Figure 5b), although a 24% decrease was observed in TSPO-KO mice. 5α-DHB concentrations were also similar in the brain (*p* = 0.3717) and testes (*p* = 0.6109) between WT and TSPO-KO animals. The concentrations of 5α-DHB were ~70 and ~200 times lower in the testes and brain, respectively, in both groups as compared to adrenal glands. In plasma, a significant 50% decrease in 5α-DHB levels was found in TSPO-KO mice (*p* = 0.0186). 3α,5α-tetrahydrocorticosterone (3α,5α-THB) was below the detection limits (<0.05 ng/g) in all tissues in both genotypes (Figure 5c). In contrast, the 3β-stereoisomer 3β,5α-tetrahydrocorticosterone (3β,5α-THB) could be measured at a high concentration in the adrenal glands of both WT and TSPO-KO mice (Figure 5d). 3β,5α-THB concentrations decreased by 31.4% in the adrenal glands of knockout mice, but no significant difference was found as compared to WT mice (Figure 5d, *p* = 0.3791). The 69% decrease in plasma in TSPO-KO mice led to a significant difference between both groups (*p* = 0.0229, Figure 5d).

#### 2.1.4. Basal Concentrations of Androgens

The androgen family in rodents is produced via the Δ4-steroid pathway starting with the synthesis of ADIONE from PROG by CYP17, which serves as an intermediate in the biosynthesis of T (Figure 1). Differences in ADIONE concentrations were not found to be statistically significant between WT and TSPO-KO mice in the brain (*p* = 0.6369), adrenal glands (*p* = 0.7964), testes (*p* = 0.9279) and plasma (*p* = 0.6336). As expected, levels of ADIONE were higher (~ten times) in the testes than adrenal glands (Table 1). Low levels of ADIONE were measured in the brain and plasma of both genotypes (Table 1).

**Figure 6:**
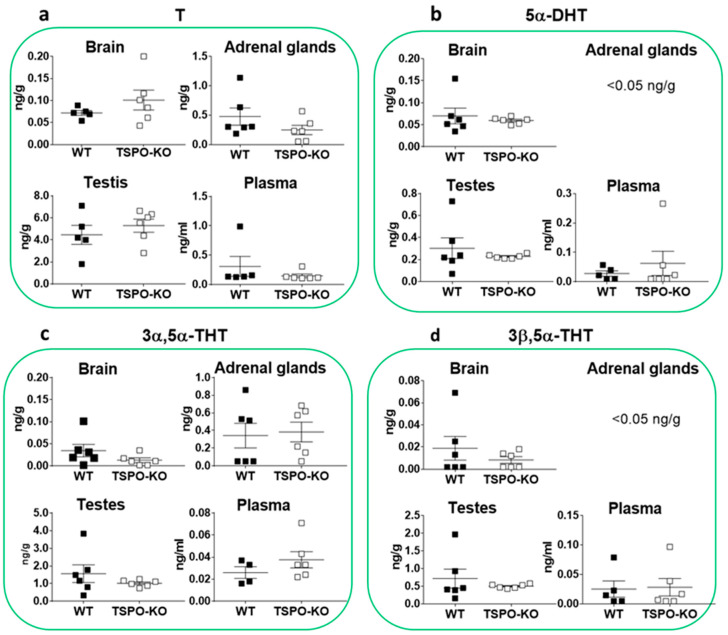
Concentrations of testosterone (T) and its 5α-reduced metabolites in tissues of male WT and TSPO-KO mice analyzed by GC-MS/MS as described in the Material and Methods section. Data are expressed as means ± SEM and were analyzed by unpaired *t*-test (*n* = 5–6).

T is the major sex steroid hormone produced in testes, with small amounts synthesized by the adrenal glands. Accordingly, in WT mice, the concentration of T was found to be eight times higher in testes than adrenal glands, but no significant difference was noted between TSPO-KO and control mice (Figure 6a, *p* = 0.4351).

As expected, the concentration of 5α-dihydrotestosterone (5α-DHT) was the highest in testes, but no significant difference was observed between WT and TSPO-KO animals (Figure 6b, *p* = 0.4448). The 3α-reduced metabolite of 5α-DHT, 3α,5α-tetrahydrotestosterone (3α,5α-THT), was also found at the highest levels in testes with no significant difference between WT and TSPO-KO (Figure 6c, *p* = 0.3054). Finally, the levels of 3β,5α-tetrahydrotestosterone (3α,5α-THT) were also comparable between both genotypes (Figure 6d, *p* = 0.437). In the adrenal glands, brain and plasma, the levels of 5α-DHT and its 3α/β-reduced metabolites were low (0.2 ng/g or ng/mL) in both groups (Figure 6b–d).

Finally, the weak androgen epiandrosterone (3β-hydroxy-5α-androstane-17-one) was detected at ~1 ng/g in adrenal glands, with no significant difference between WT and TSPO-KO mice (Table 1). Very low and comparable levels of androstenediol (androst-5-ene-3β,17βdiol, ADIOL), a precursor of T, could be detected in the testes and brain in both genotypes (Table 1).

### 2.2. Basal Protein Levels of TSPO/PBR, VDAC-1, CYP11A1 and 5α-Reductase (SRD5A)

#### 2.2.1. TSPO/PBR Protein Levels

**Figure 7:**
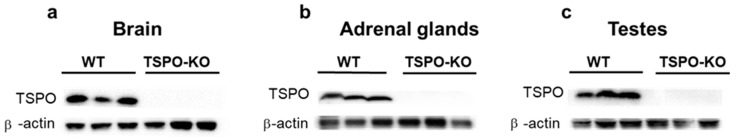
Western blot analysis of TSPO/PBR in tissues from male WT and TSPO-KO mice. Representative bands of TSPO/PBR and the loading control β-actin for the brain (**a**), adrenal glands (**b**) and testes (**c**) are shown.

We previously demonstrated that TSPO/PBR protein was undetectable in lysates from the kidney, spleen and testes in TSPO-KO mice [19]. Here, we showed that TSPO/PBR was completely absent in the brain and adrenal glands of TSPO-KO mice, while confirming its absence in the testes, as compared to WT mice (Figure 7).

#### 2.2.2. VDAC-1 Protein Levels

**Figure 8:**
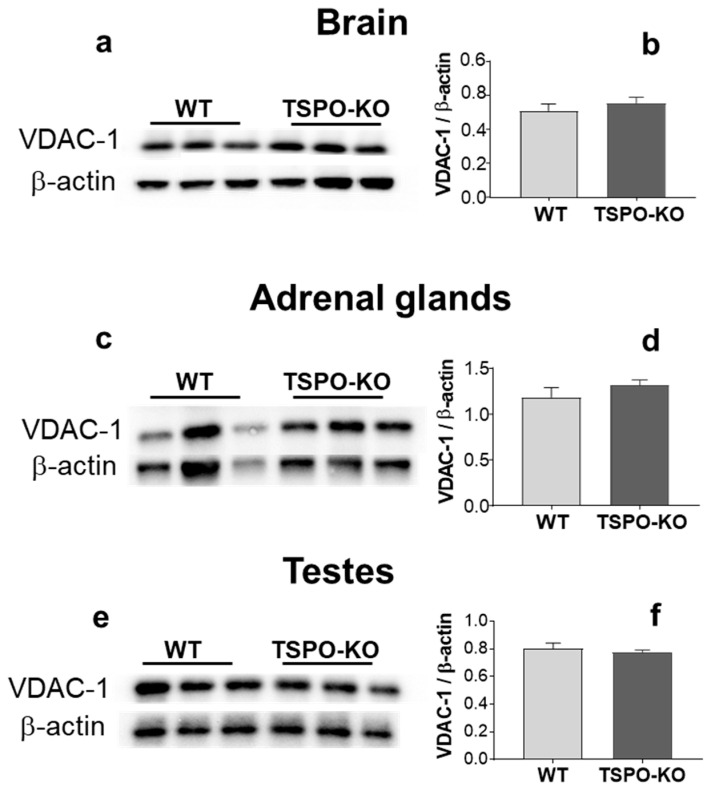
Western blot analysis of VDAC-1 protein levels in the brain, adrenal glands and testes from TSPO-KO and WT mice. Representative bands of VDAC-1 and the loading controls β-actin or GAPDH for all tissues (**a**,**c**,**e**) are shown. Quantification of VDAC-1 levels was normalized to the loading control β-actin (**b**,**d**,**f**).

A comparison of VDAC-1 levels between WT and TSPO-KO mouse tissues did not show any significant changes (Figure 8). Indeed, basal levels of VDAC-1 were similar in both groups of mice in the brain (Figure 8a,b; *p* = 0.4623), adrenal glands (Figure 8c,d; *p* = 0.3534) and testes (Figure 8e,f; *p* = 0.5849).

#### 2.2.3. CYP11A1 Protein Levels

**Figure 9:**
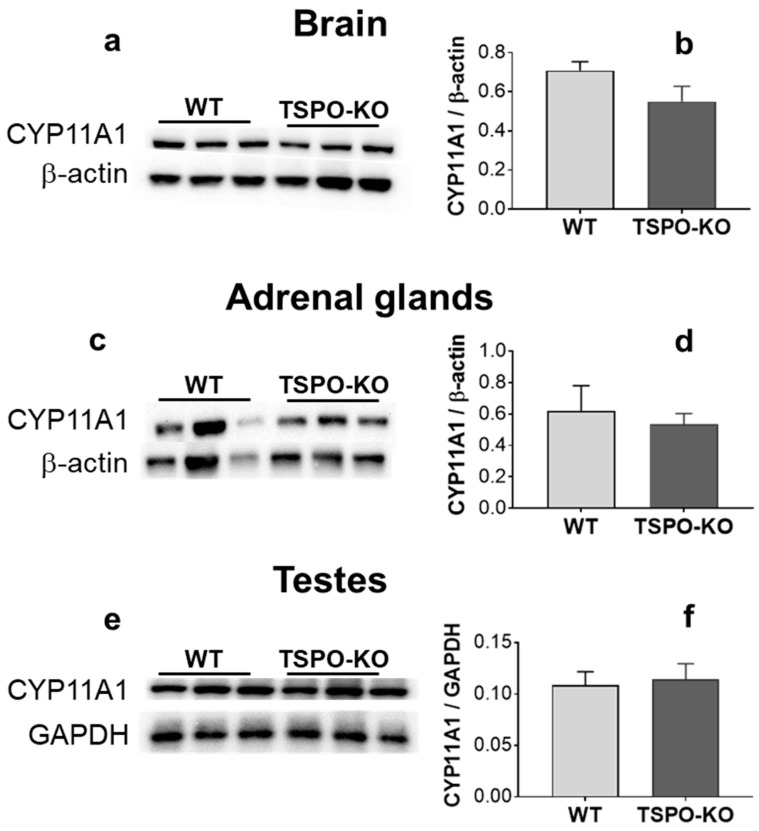
Western blot analysis of CYP11A1 protein levels in the brain, adrenal glands and testes from TSPO-KO and WT mice. Representative bands of VDAC-1 and the loading controls β-actin or GAPDH for all tissues are shown (**a**,**c**,**e**). Quantification of CYP11A1 levels was normalized to the loading control (**b**,**d**,**f**).

Whether the lack of TSPO/PBR has an impact on CYP11A1 levels was investigated in the brain, adrenal glands and testes in comparison to WT controls. The results shown in Figure 8 revealed that the levels of CYP11A1 were not significantly different across genotypes in the brain (despite the 22% decrease in the TSPO-KO mice; Figure 9a,b, *p* = 0.156), adrenal glands (Figure 9c,d, *p* = 0.643) and testes (Figure 9e,f, *p* = 0.793).

#### 2.2.4. 5α-Reductase (SRD5A) Protein Levels

**Figure 10:**
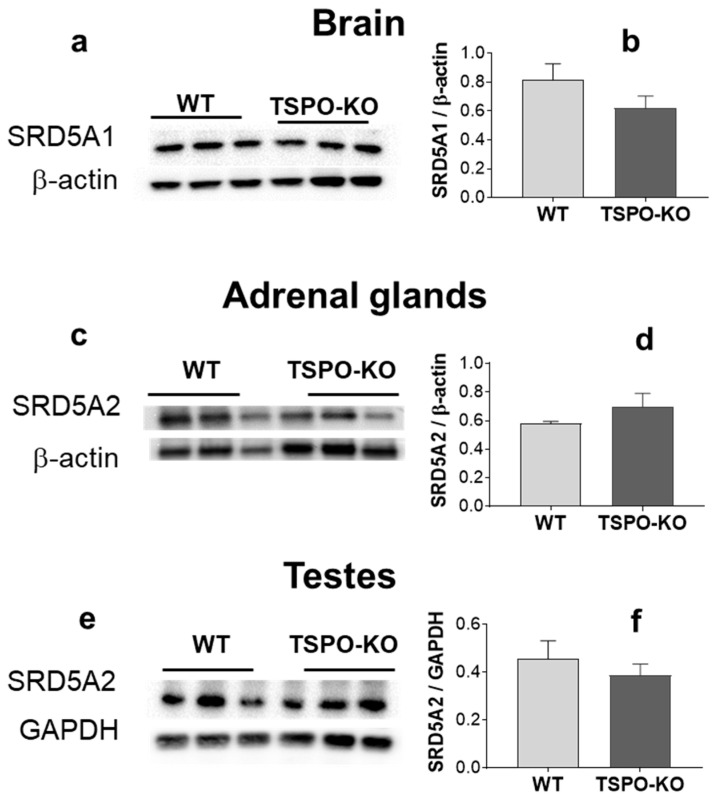
Western blot analysis of SRD5A protein levels in the brain, adrenal glands and testes from TSPO-KO and WT mice. Representative bands of SRD5A1, SRD5A2 and the loading controls β-actin or GAPDH for all tissues are shown (**a**,**c**,**e**). Quantification of SRD5A levels was normalized to the loading control (**b**,**d**,**f**).

Two 5α-reductase steroids, designated type 1 (SRD5A1) and type 2 (SRD5A2), have been identified in rodents and humans [30,31]. SRD5A1 is most abundant in brain [31,32] and SRD5A2 is more strongly expressed in peripheral tissues [33]. The basal level of SRD5A1 in the brain of TSPO-KO mice was not significantly different from that in WT mice (Figure 10a, *p* = 0.237). The basal levels of SRD5A2 in TSPO-KO mice were also not significantly different in adrenal glands (Figure 10b, *p* = 02856) and testes (Figure 10c, *p* = 0.474) as compared to WT mice.

## 3. Discussion

Our detailed steroidome in complete C57BL/6-^Tspotm1GuWu(GuwiyangWurra)^-knockout male mice (TSPO-KO) addressed the fundamental question of whether the ancient evolutionarily conserved pbr/tspo gene is essential with respect to steroidogenesis in vivo in mice. To our knowledge, this is the first study providing a steroidome in vivo in complete TSPO-KO mice using a specific, reliable, sensitive and well-validated procedure based on GC-MS/MS. We were able to analyze and quantify simultaneously 23 steroids in individual brains, adrenal glands, testes and plasma.

We used complete C57BL/6-^Tspotm1GuWu(GuwiyangWurra)^-knockout male mice (TSPO-KO that we previously created. Long-term observations have shown that these TSPO-KO mice have normal lifespans and fertility and unimpaired cholesterol transport [19]. Our data show that steroidogenesis appeared unaltered in the main classical steroidogenic organs, despite the absence of TSPO/PBR. Indeed, PREG, the steroid produced directly from cholesterol in mitochondria, was present in all tissues of TSPO-KO mice at unchanged levels relative to WT mice, thus clearly indicating that TSPO/PBR was not required for PREG synthesis. This result supported our previous findings in the same TSPO-KO model showing no significant difference in PREG levels in serum [19]. Barron et al. [25] have also reported unchanged circulating levels of PREG in global TSPO-KO mice. In the present work, we also found no alteration in CYP11A1 protein levels in all tissues tested, regardless of genotypes.

If TSPO/PBR does not have an impact on PREG levels, any change in downstream metabolic pathways is unrelated to de novo steroidogenesis. In male rodents, PREG is preferentially metabolized to PROG by the 3β-HSD type I [34]. PROG is a key steroid hormone that can be metabolized to two other main steroid hormones, B in adrenal glands and T in testes. Here, the concentration of PROG in the adrenal glands of TSPO-KO mice was not statistically different from that in WT mice, again suggesting that deletion of the tspo gene also does not affect the conversion of PREG to PROG. This result contrasts with the significant reduction in circulating PROG in global TSPO-KO mice observed by Barron et al. [25] while circulating PREG remained unchanged. As for PROG, we did not find significant changes in any tissues for B concentrations due to the absence of TSPO/PBR. Barron et al. [25] found a significant decrease in serum B in global TSPO-KO mice and Fan et al. [13] reported unchanged B levels in response to adrenocorticotropic hormone in conditional TSPO-KO mice. These discrepancies cannot be explained by the C57BL/6 genetic background of the mice because C57BL/6 mice were used in all studies. We also found that the levels of T and its precursor ADIONE were also unaltered in TSPO-KO mice. Fan et al. [13] reported increased circulating testosterone levels in conditional TSPO-KO mice following human chorionic gonadotropin stimulation. Thus, the biosynthesis of adrenal and testicular steroid hormones is not critically dependent on TSPO/PBR.

The identification and quantification of the 5α-reduced derivatives of PROG, T, DOC, B and ADIONE in the TSPO-KO animals are the new findings in the present study. To our knowledge, these endogenous 5α-dihydro-3-keto derivatives have never been analyzed in any tissue after deletion of the tspo/gene. Steroid 5α-reductases catalyze an irreversible reduction of the double bond between carbons four and five of these steroids. This enzymatic reaction involves the cofactor nicotinamide adenine dinucleotide phosphate (NADPH), a high-energy electron carrier that transports energy within cells and which becomes NADP+ (lower energy cofactor) once its electrons have been released during reduction.

We found significant reductions in the concentrations of 5α-DHP in the adrenal glands and plasma and of 5α-DHB in the plasma of TSPO-KO relative to WT mice that could not be explained by any change in the levels of their precursors, PROG and B, respectively, which remain unchanged after tspo gene deletion. Therefore, the decrease in the 5α-reductase activity suggests a regulatory effect of TSPO/PBR on a different mitochondrial or cell function.

Based on phylogenetic considerations, the TSPO/PBR can be linked to oxygen sensing, respiratory control ATP synthesis and, thus, energy metabolism as a function that evolved early in evolution [35,36]. Indeed, TSPO/PBR has been shown to play an important role in cellular bioenergetics [37,38,39,40], and reports have shown a correlation between high TSPO/PBR expression and tissues deriving their metabolic energy from oxidative phosphorylation, such as the adrenal cortex and skin [41]. Accordingly, the complete absence of TSPO/PBR in our C57BL/6-^Tspotm1GuWu(GuwiyangWurra)^-knockout model, despite the absence of an overtly abnormal phenotype, resulted in decreased mitochondrial membrane potential and ATP production [20], suggesting reduced energy metabolism. Steroid 5α-reductase isozymes require NADPH as a cofactor, and their activity is linked to the availability of ATP and mitochondrial respiration [42,43,44]. Accordingly, it has been suggested that 5α-reductase activity may be energy dependent and diminished by dephosphorylating agents [45].

The present study is also the first to systematically test for potential variations in the concentrations of the 3α- and 3β,5α-reduced metabolites of PROG, DOC, B and T. Our data indicate a reduction in the concentration of adrenal 3α,5α-THPROG and plasmatic 3β,5α-THB (the 3α,5α-reduced metabolite of PROG and the 3β,5α-reduced metabolite of B) in TSPO-KO mice as compared to wild-type animals, which might be explained by decreased levels of their precursors 5α-DHP and 5α-DHB, respectively. It is noteworthy that complete deletion of the Tspo gene did not alter the levels of 5α-DHT and its 3α/β-reduced derivatives, regardless of the tissue. Collectively, these data suggest that the modulation of specific 5α- and 3α/β-reduced metabolites of PROG and B is not linked to de novo steroid synthesis. Given that there is no known structure–function link between TSPO/PBR and enzymatic activities of SRD5A and 3α/3β-HSOR, we interpreted this effect of TSPO/PBR on 5α-reductase activity as evidence for a role of TSPO/PBR in another mitochondrial or cell metabolic process, such as the regulation of basic cellular energy metabolism, as discussed earlier.

In summary, our work provides comprehensive evidence that de novo steroidogenesis does not critically depend on the presence of TSPO/PBR. Our results corroborate the findings of previous reports from independent groups [19,21,22,23] indicating that TSPO is not involved in the de novo steroidogenesis, and disagree with prior reports suggesting TSPO-dependent steroidogenesis [6,12,13,25,46]. Notably, TSPO/PBR may regulate intracellular cholesterol transport through mechanisms not necessarily related to steroid biosynthesis [47].

## 4. Conclusions/Open Questions

The nomenclature for the peripheral benzodiazepine receptor (PBR), an earlier operational definition of the protein based on non-GABA_A_ receptor binding by diazepam [41], was changed in 2006 to “18 kDa translocator protein” due to its postulated indispensable role in the translocation of cholesterol across the outer mitochondrial membrane [48]. However, our comprehensive analysis of steroidogenesis in the absence of TSPO/PBR failed to substantiate an obligatory role of TSPO/PBR-dependent cholesterol translocation in steroidogenesis. In view of our results and other earlier observations, notably with regard to the likely broader bioenergetic functions that appear to depend on the presence of TSPO/PBR, there is a need to revisit our understanding of the originally posited molecular functions that led to the renaming of the PBR.

With regard to the functional mechanisms of TSPO/PBR, the following issues merit more detailed investigation and verification:

(1) Is there sufficient evidence to identify cholesterol translocation as the main function of the TSPO/PBR?
(2) Does PREG synthesis involve enzymes other than mitochondrial CYP11A1, as recently suggested in human glial cells [49], or autooxidation pathways that are independent from mitochondrial cholesterol?
(3) Could TSPO knockout alter the biophysical properties of the outer and inner membranes of mitochondria [50,51] and, in particular, lead to a reorganization of the proteins involved in energy production?
(4) While steroid concentrations in the adrenal glands, testes, brain and plasma appeared to be largely unchanged after a complete deletion of the tspo gene, there was a significant decrease in some 5α-reduced steroids in adrenal glands, which requires further investigation. Is the change in 5α-reductase enzymatic activity biologically significant?
(5) Finally, in light of the above issues, should a conceptually more neutral, new nomenclature be used instead of TSPO—a naming according to a purported, no longer adequately substantiated main function—or PBR—an historical definition based on the affinity seen for certain benzodiazepines? In the meantime, one could consider the combined term “TSPO/PBR” in order to retain the unity of knowledge about PBR achieved before the name change in 2006 and the multiple perspectives on the putative function of the protein.

Despite controversy over the role of TSPO/PBR in de novo steroidogenesis and residual questions to be solved, TSPO/PBR remains a protein of significant relevance for drug development. It binds multiple classes of molecules, performs several functions and participates in essential mitochondrial physiological processes [40]. Moreover, it has become a biomarker and a potential therapeutic drug target for neuropsychiatric, neurodegenerative, inflammatory diseases [52,53,54,55], and a broadened view on the function(s) of TSPO/PBR should rekindle or accelerate progress in the clinical development of existing and new drugs.

## 5. Materials and Methods

### 5.1. Ethics

All animal procedures were carried out in accordance with the European Community Council Directive of 2010/63/EU for the care and use of laboratory animals for scientific purposes (animal facility authorization number: D 94-043-013). They were approved by the French Ministry of National Education, Higher Education, Research and Innovation after ethical approval from the local Ethics Committee on Animal Experimentation (registration under APAFIS#1000462017052219143381v3).

### 5.2. Animals

Complete Tspo knockout mice named C57BL/6-Tspotm1GuWu (GuwiyangWurra)-knockout mice (TSPO-KO) were generated as previously described [19]. Briefly, the targeting construct containing loxP sites flanking exons 2 and 3 and a neomycin cassette between exons 3 and 4 was electroplated into C57BL/6 embryonic stem cells. Cells that were correctly targeted were injected into C57BL/6 mice. Mice positive for the presence of targeted Tspo alleles were crossed with C57BL/6 Cre-deleted mice to generate heterozygous global Tspo-deficient mice, which were then bred with wild-type C57BL/6 mice to remove the Cre transgene. Mice were genotyped by Southern blot using genomic DNA isolated from tail biopsies and amplified by PCR.

The male Tspo knockout mice (TSPO-KO) and wild-type controls (WT, *n* = 9) were 5 months old and their average weights were 30.3 ± 0.6 g (*n* = 9) and 27.6 ± 0.7 g (*n* = 9), respectively. They were kept in ventilated racks housing five mice per cage containing a constant supply of food pellets and tap water and enriched with crimp brown Kraft and wooden sticks. All animals were maintained under controlled animal house conditions (21 ± 2 °C; hygrometry: 55 ± 5%; 12 h light/dark cycle, lights at 7:00 a.m.). Prior to the start of experiments, mice had a 2 week habituation/handling period with the principal authorized investigator.

### 5.3. Experimental Designs

Naive WT and TSPO-KO mice were used. Mice were euthanized by rapid decapitation without anesthesia. Blood was collected in heparinized plastic tubes and plasma (200–400 µL) was prepared by centrifugation at 4 °C and stored at −80 °C. Forebrains (with olfactory bulbs), testes and adrenal glands were removed, quickly frozen in liquid nitrogen, weighed and stored at −80 °C.

Steroid profiling was performed for the brain, adrenal glands and testes of WT and TSPO-KO mice (*n* = 6/group) by GC-MS/MS. In addition, the basal levels of TSPO, VDAC-1, CYP11A1 and SRD5A were analyzed in the brain, adrenal glands and testes of WT and TSPO-KO mice with Western blotting (*n* = 3/group).

### 5.4. Steroid Profiling by Gas Chromatography Coupled to Tandem Mass Spectrometry (GC-MS/MS)

A whole panel of steroids was identified and quantified simultaneously in individual tissues with a GC-MS/MS procedure fully validated in terms of accuracy, reproducibility and linearity for nervous tissue and plasma, which has been previously described [26]. Briefly, the following internal standards were added into the tissue methanolic extracts for steroid quantification: 2 ng of ^13^C_3_-PROG for PROG; 2 ng of ^2^H_6_-5α-DHP (CDN Isotopes, Sainte-Foy-la-Grande, France) for 5α/β-DHP and 5α/β-DHADIONE; 2 ng of epietiocholanolone (3β-hydroxy-5β-androstan-17-one, Steraloids, Newport, Rhode Island) for androstenediol (ADIOL), epiandrosterone, 5α/β-dihydrotestosterone (DHT), 3α/β5α/β-tetrahydrotestosterone (THT), pregnenolone (PREG), 20α-dihydropregnenolone (DHPREG), 3α/β5α/β-tetrahydroprogesterone (THPROG), 5α/β20α-THPROG, 3α/β5α/β20α/β-hexahydroprogesterone (HHPROG) and 3α/β5α/β-tetrahydrodeoxycorticosterone (THDOC); 2 ng of ^13^C_5_-20α-dihydroprogesterone (DHP) for 20α/β-DHP; 2 ng of 19-norPROG for 5α/β-dihydrodeoxycorticosterone (5α/β-DHDOC); 10 ng of ^2^H_8_-corticosterone (B) for B, 5α/β-dihydrocorticosterone (DHB), 3α/β5α/β-tetrahydrocorticosterone (THB) and 11-dehydrocorticosterone (11-dehydroB); 2 ng of ^13^C_3_-deoxycorticosterone (DOC) for DOC; 1 ng of ^13^C_3_-androstenedione (ADIONE) for ADIONE; 1 ng of ^13^C_3_-testosterone (T) for T; and 1 ng of ^2^H_5_-estradiol (E2) for E2 and 20α/β-dihydroprogesterone (20α/β-DHP).

Extracts were then purified and fractionated by solid-phase extraction with the recycling procedure [56]. The unconjugated steroid-containing fraction was filtered and fractionated with an HPLC system composed of a WPS-3000SL analytical autosampler, a LPG-3400SD quaternary pump gradient coupled with a SR-3000 fraction collector (ThermoFisher scientific, San Jose, CA, USA) and a Lichrosorb Diol column (25 cm, 4.6 mm, 5 µm) in a thermostated block at 30 °C. The column was equilibrated in a solvent system of 90% heptane and 10% of a mixture composed of heptane/isopropanol (85/15). Elution was performed at a flow rate of 1 mL/min, first with 90% heptane and 10% heptane/isopropanol (85/15) for 15 min, then with a linear gradient to 100% acetone in 2 min. This mobile phase was kept constant for 13 min.

Two fractions were collected from the HPLC system: 5α/β-DHPROG and 5α/β-DHADIONE were eluted in the first HPLC fraction (3–15 min) and then silylated with 50 μL of a mixture of N-methyl-N-trimethylsilyltrifluoroacetamide/ammonium iodide/dithioerythritol (1000:2:5) (vol/wt/wt) for 15 min at 70 °C. The second fraction (15–29 min) containing all other steroids was derivatized with 25 μL heptafluorobutyric anhydride (HFBA) and 25 μL anhydrous acetone for 1 h at 20 °C. Both fractions were dried under a stream of N2 and resuspended in heptane.

GC-MS/MS analysis of the purified and derivatized extracts was performed using an AI1310 autosampler, a Trace 1310 gas chromatograph (GC) and a TSQ8000 mass spectrometer (MS) (Thermoscientific, San Jose, CA, USA). Injection was performed in the splitless mode at 250 °C (1 min of splitless time) and the temperature of the gas chromatograph oven was initially maintained at 80 °C for 1 min and ramped between 80 and 200 °C at 20 °C/min, then ramped to 300 °C at 5 °C/min and finally ramped to 350 °C at 30 °C/min. The helium carrier gas flow was maintained constant at 1 mL/min during the analysis. The transfer line and ionization chamber temperatures were 330 °C and 200 °C, respectively. Electron impact ionization was used for mass spectrometry with an ionization energy of 70 eV. GC-MS/MS signals were evaluated using a computer workstation by means of the software Excalibur^®^, release 3.0 (ThermoFisher scientific, San Jose, CA, USA). Identification of steroids was supported by their retention time and according to two or three transitions. Quantification was performed according to the transition giving the more abundant product ion. The range of the limit of detection was roughly 0.5–10 pg according to the steroid structure.

The analytical protocol has been validated for all the targeted steroids using extracts of 200 mg of a pool of male mouse brains. The evaluation included the limit of detection, linearity, accuracy and intra-assay and inter-assay precision [26].

### 5.5. Western Blot

Individual brains, adrenal glands, testes and liver from naive WT and TSPO-KO mice were quickly weighed, homogenized in tenfold volume cold RIPA lysis buffer (Santa Cruz) containing 0.1% protease cocktail inhibitors (Sigma, Saint-Louis, MO, USA) and briefly sonicated (Brandon Sonifier cell disrupter). Tissue homogenate was centrifuged at 13,000× *g* for 15 min at 4 °C to remove insoluble material. An aliquot of the resulting supernatant (lysate) was used to determine the amount of proteins in the BCA protein assay (Thermoscientific, Rockford, IL, USA) and the remainder was stored at −80 °C. Equal numbers of proteins (30 µg) were mixed in one-fifth volume Laemmli sample buffer, heated at 95 °C for 5 min, loaded into a precast 10% acrylamide Mini-Protean TGX gel (Bio-Rad, Hercules, CA, USA) together with 10 µL of ECL Rainbow marker full range (Amersham) and separated by electrophoresis in Tris-Glycine-Sodium Dodecyl Sulfate Running Buffer 1X (Bio-Rad). The proteins were then transferred using a Mini Transblot Turbo (Bio-Rad) onto a polyvinylidene fluoride membrane (Biorad). The membranes were incubated in 5% nonfat dry milk with 0.1% tween-20 in PBS 1x for 1 h to block non-specific binding sites. They were then incubated with the primary antibodies diluted in blocking solution overnight at 4 °C with mild shaking, followed by 1 h incubation with horseradish peroxidase-labeled anti-rabbit or anti-mouse secondary antibodies (Biorad). Immunoreactive bands were detected using the enhanced ECL Radiance or Radiance Q (Azure Biosystems, Dublin, CA, USA). Protein density was revealed with the Bio-Rad blot imaging system ChemiDoc XRS+, which was set up to reveal saturated pixels. Image J software was used to quantify non-saturated bands. Levels of individual proteins were normalized to the levels of β-actin or GAPDH from the same sample. Antibodies used were as follows: rabbit anti-PBR (abcam), mouse anti-CYP11A1 (abcam), rabbit anti-5α-reductase type 1 (SRDA5A1, Thermofisher scientific, Waltham, MA, USA), rabbit anti-5α-reductase type 2 (SRDA5A2, ABclonal), rabbit anti-VDAC1 (Millipore, Temecula, CA, USA), mouse anti-β-actin (AC74, Sigma Aldrich) and mouse anti-GAPDH (Millipore).

### 5.6. Statistical Analysis

Data were expressed as the means ± SEM. They were processed using GraphPad Prism 7.0 (GraphPad Inc, San Diego, CA, USA). When the Shapiro–Wilk test was applied on steroid concentrations and protein levels, p values were larger than 0.05 in all groups, and we therefore assume a normal distribution. The differences between the two groups were analyzed with the unpaired two-tailed Student’s *t*-test. *p* values < 0.05 were considered statistically significant. In a very few cases, a value for a specific steroid was missing from the dataset due to a problem encountered during the GC-MS/MS procedures or an outlier eliminated using the Grubbs’s Q test.

## Figures and Tables

**Table 1:** Basal concentrations of 20α-reduced steroids, ADIONE, epiandrosterone and androstenediol in tissues of male WT and TSPO-KO mice (*n* = 5–6).

Basal Steroid Concentrations	Brain (ng/g)			Adrenal Glands (ng/g)			Testes (ng/g)			Plasma (ng/mL)		
	WT	TSPO-KO	*p*	WT	TSPO-KO	*p*	WT	TSPO-KO	*p*	WT	TSPO-KO	*p*
**20α-DHPREG**	0.41 ± 0.08	0.36 ± 0.04	*0.590*	0.32 ± 0.06	0.27 ± 0.08	*0.627*	<0.05	<0.05	*------*	0.021 ± 0.00	0.02 ± 0.00	*0.948*
**20α-DHP**	0.06 ± 0.01	0.06 ± 0.02	*0.989*	0.09 ± 0.02	0.10 ± 0.03	*0.906*	0.09 ± 0.02	0.08 ± 0.01	*0.745*	0.03 ± 0.01	0.02 ± 0.01	*0.348*
**5α,20α-THP**	1.13 ± 0.15	1.11 ±0.22	*0.959*	0.72 ± 0.15	1.23 ± 0.36	*0.218*	0.05 ± 0.01	0.05 ± 0.01	*>0.99*	0.12 ± 0.03	0.10 ± 0.02	*0.500*
**3α,5α,20α-HHP**	0.55 ± 0.06	0.69 ± 0.11	*0.600*	1.63 ±0.04	1.63 ± 0.51	*>0.999*	0.24 ± 0.06	0.32 ± 0.11	*0.560*	0.33 ± 0.04	0.27 ± 0.05	*0.335*
**3β,5α,20α-HHP**	0.11 ± 0.01	0.15 ±0.05	*0.457*	2. 55 ± 0.35	2.61 ± 0.77	*0.943*	0.05 ± 0.01	0.05 ± 0.01	*>0.999*	0.04 ± 0.01	0.05 ± 0.01	*0.708*
**ADIONE**	0.11 ± 0.05	0.09 ± 0.01	0.637	2.8 ± 0.7	3.0 ± 0.4	0.796	31 ± 7	31 ± 3	0.928	0.12 ± 0.02	0.11 ± 0.01	0.634
**Epiandrosterone**	<0.05	<0.05	------	1.31 ± 0.27	1.00 ± 0.36	0.506	0.11 ± 0.04	0.07 ± 0.01	0.354	0.01 ± 0.00	0.01 ± 0.00	>0.99
**Androstenediol**	0.10 ± 0.01	0.11 ± 0.04	0.776	<0.005	<0.005	------	0.07 ± 0.03	0.06 ± 0.01	0.647	<0.05	<0.05	------

## Data Availability

The original contributions developed in the study are present in the article.
